# Gastrointestinal hemorrhage caused by small intestinal benign tumors: 2 cases report

**DOI:** 10.11604/pamj.2019.33.197.18584

**Published:** 2019-07-12

**Authors:** Mohamed Kane, Mohamed Zakaria Benaboud, Samba Traore, Salma Lokman, Soumia Nasri, Narjisse Aichouni, Imane Skiker, Imane Kamaoui

**Affiliations:** 1 Radiology Department of Mohammed VI hospital, Oujda, Morocco

**Keywords:** Small intestinal lipoma, gastrointestinal bleeding, computed tomography angiography

## Abstract

Gastrointestinal bleedings caused by small intestinal tumors are rare and difficult to diagnose because they are not easy to access to the conventional endoscopy. We report two cases, one of them from proximal jejunum and the other one from ileal intestine complicated by intussusception. The two cases were admitted in the emergency department for hematochezia and melena, the diagnosis was established by enhanced helical computed tomography angiography.

## Introduction

Jejuno ileal tumors are uncommon causes of gastrointestinal bleeding (GIB); they represent less than 5% of all causes of GI hemorrhage [1, 2]. Gastrointestinal lipomas are benign, usually single, slow growing, nonepithelial tumors. The most common site is the colon, although they may also be found in the stomach, esophagus, and small intestine [3, 4]. The diagnosis of these pathologies is almost difficult. It was made thanks to the realization of an abdominal helical computed tomography angiography (CTA). We describe two cases of gastrointestinal bleeding due to small intestinal tumors.

## Patient and observation

### Case 1

**Figure 1:**
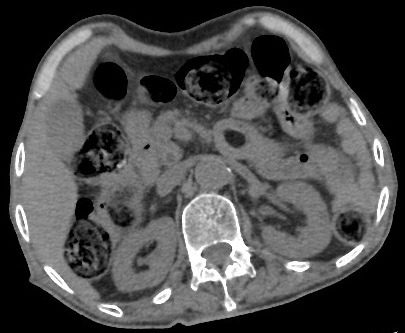
Abdominal non enhanced helical CT. Transverse axial scan showing an oval hypodense lesion typical of fatty tissue, surrounded by the wall of proximal jejunum

**Figure 2:**
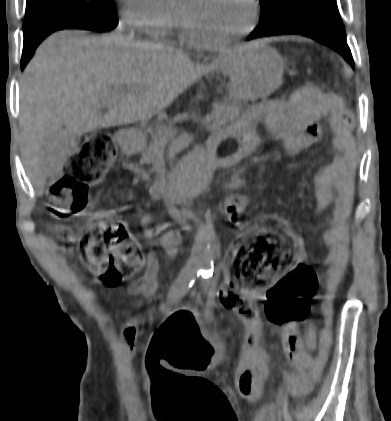
Coronal scan showing a lipoma in the wall of jejunum intestine

A 72-year-old male without pathological antecedent went to the emergency room with asthenia and melena. On examination, he presented tachycardia. His blood tests showed microcytic hypochromic anaemia (haemoglobin 6.7 g/dL). After receiving 2 Units of red blood cells, the patient underwent upper endoscopy, which was unremarkable. An abdominopelvic CT scan with helical cuts at intravenous bolus time (4 mL/s) was performed. The examination showed, in the wall of the jejunum, a low-density tissue formation (-70 HU), not enhanced after injection of the contrast medium. This image was compatible with a tumor evoking a lipoma (Figure 1, Figure 2)

### Case 2

**Figure 3:**
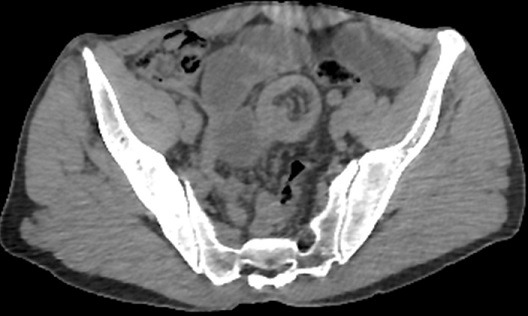
Abdominal non-enhanced helical CT. Transverse axial scan showing the tortuous intestine and its mesentery in the middle low abdomen with a ring-shaped appearance the target sign of the intestinal intussusception

**Figure 4:**
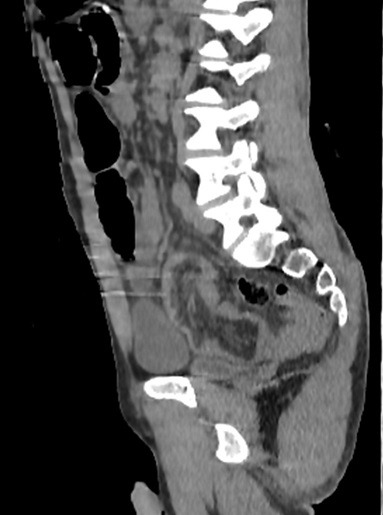
Coronal scan showing a lipoma in the wall of jejunum intestinelongitudinal reconstruction of non-enhanced abdominal CT showing the congestion of mesenteric vessels in the tortuous intestine

A 68 years old male with a past history of arterial hypertension, hyperlipidemia, and type 2 diabetes treated with oral therapy, presented with hematochezia. On physical examination, he was pale, with a temperature of 37.1°C, a pulse rate of 85 beats per minute, a blood pressure of 140/83 mmHg, and a respiration rate of 20 breaths per minute. A rectal examination revealed hematochezia. Laboratory tests were indicative of normocytic normochromic anemia (haemoglobin 8.2g/dL), after receiving 2 Units of red blood cells, the patient underwent to rectosigmoidoscopy which was featurless. In the following an abdominal enhanced helical computed tomography angiography was performed which revealed a target sign with a central component with adipose tissue in the ileal portion of small intestine (Figure 3, Figure 4). A biopsy and endoscopic treatment were not indicated due to the large size and wide stalk of the lesion, which were considered to increase the risk of active bleeding and perforation. Our patient underwent laparoscopic surgery of his small intestine (segmental resection). A pathological examination of the resected tumor showed a pedunculated lipoma of the ileum.

## Discussion

Gastrointestinal bleeding (GIB) caused by small intestinal tumors is a very rare entity that accounts for less than 5% of all cases of gastrointestinal GIB [3, 5]. Tumors of the small intestine are cited among the causes of obscure gastrointestinal bleeding before the age of 50 years. Zhang *et al*. [3, 6] reported that the common causes of small intestinal bleeding were: vascular anomalies (54.35%), small intestinal ulcer (13.04%) and small intestinal tumors (11.96%) in older patients (>65 years); vascular anomalies (34.82%), small intestinal tumors (31.25%), nonspecific enteritis (9.82%) in middle age (41-64 years); Crohn's disease (34.55%), small intestinal tumors (23.64%) and nonspecific enteritis (10.91%) in young adults (<40 years) [6, 7]. Primary lipomas of the small intestine are also unusual, representing 2.6% of nonmalignant tumors of the intestinal tract [8]. The most common location is the colon (65 to 70%), followed by the ileum and jejunum (20 to 25%) and rarely the esophagus and stomach [3]. They are rarely manifested by gastrointestinal bleeding. At present, only a few cases have been reported in the literature. Lipoma is a benign tumor that can develop throughout the human body. Its intestinal localization was first described in 1818 by Meckel. The ileum accounts for about 60% of all lipoma localizations in the small intestine. It is represented by a mass of fatty tissue circumscribed by a fibrous capsule. Its topography is often submucosal, sometimes subserosal. Small intestinal lipomas have a rounded shape and are frequently pedunculated, asymptomatic in most of cases. They can cause abdominal pain, transit disorders, or GIB. The most common complication is ileal-ileal intussusception. They may even have a sub acute or even chronic evolution by spontaneous disinvagination. Necrosis, ulceration and gastrointestinal bleeding are more rare complications. Their degeneration is quite exceptional.

In the present report, the two patients both underwent abdominal CT examinations after endoscopic explorations which were unremarkable. The abdominal CT showed in both cases a small intestinal regular tumor, smooth, round, well-demarcated lesions, sometimes pedunculated with a coefficient of fat attenuation, complicated by intestinal intussusception in the second case. Our study suggested if the diagnosis of the cause of gastrointestinal bleeding was difficult in endoscopy, abdominal CTA can be chosen as the next step. On endoscopic examination, lipomas tend to appear as yellowish, smooth, round or hemispherical tumors, with either a pedunculated or wide stalk. The cushion sign and naked fat sign are also specific to lipomas [3, 9]. Sawada *et al*. [10] and Chou *et al*. [9] reported that small intestinal lipomas causing OGIB were characterized by a wide stalk and ulceration. In contrast, in the present case, the small intestinal tumor was whitish, pedunculated, and cylindrical, with ulceration on the top. Endoscopic investigation did not allow conclusive diagnosis of a typical small intestinal lipoma. Endoscopists should be aware of small intestinal lipomas that exhibit a whitish color and cylindrical shape [3]. Histopathological findings revealed that the submucosal lipoma consisted of mature fat [3]. Benign small intestinal tumors are treated according to their clinical symptoms and size. If a patient with small bowel lipomas is asymptomatic, supportive treatment is generally recommended. However, the mainstay treatment of patients with symptomatic small intestinal tumors is surgery. The endoscopic treatment of patients with symptomatic small bowel lipoma is an alternative if small bowel lipoma can be removed by endoscopic resection [11].

## Conclusion

In conclusion, small intestinal lipomas are rare, cause of GI bleeding. The abdominal CTA is a good modality for the diagnosis of these pathologies after the failure of endoscopic explorations.

## Competing interests

The authors declare no competing interests.
